# Exploring Global Interest in Propolis, Nanosilver, and Biomaterials: Insights and Implications for Dentistry from Big Data Analytics

**DOI:** 10.3390/dj13060253

**Published:** 2025-06-06

**Authors:** Magdalena Sycińska-Dziarnowska, Liliana Szyszka-Sommerfeld, Krzysztof Woźniak, Gianrico Spagnuolo

**Affiliations:** 1 Department of Maxillofacial Orthopaedics and Orthodontics, Pomeranian Medical University in Szczecin, Al. Powst. Wlkp. 72, 70111 Szczecin, Poland; 2 Laboratory for Propaedeutics of Orthodontics and Facial Congenital Defects, Pomeranian Medical University in Szczecin, 70111 Szczecin, Poland; 3 Department of Neurosciences, Reproductive and Odontostomatological Sciences, University of Naples “Federico II”, 80131 Napoli, Italy; 4 School of Dentistry, College of Dental Medicine, Kaohsiung Medical University, Kaohsiung 80708, Taiwan

**Keywords:** nanosilver, propolis, antibacterial, antimicrobial, Google Trends

## Abstract

**Background**: The growing demand for innovative biomaterials with antimicrobial properties has driven research into natural and synthetic compounds, such as propolis and nanosilver, known for their antimicrobial efficacy. **Methods**: This study uses Google Trends data to analyze global search interest in five key terms—*propolis*, *antimicrobial*, *antibacterial*, *nanosilver*, and *biomaterials*—over a ten-year period (starting November 2014). The objective is to evaluate temporal variations, quantify correlations between the terms, and explore how external events, such as the COVID-19 pandemic, have influenced public and clinical interest in these topics. Search data were extracted, normalized, and analyzed using multivariate time series methods, including vector autoregression (VAR) modeling, Impulse Response Function (IRF) analysis, and forecast error variance decomposition (FEVD). Stability, causality, and inter-period relationships were assessed using statistical analysis, with results visualized through time series plots and impulse response coefficients. **Results**: Key findings reveal significant interdependencies between search terms, with surges in one often resulting in immediate or short-term increases in others. Notable trends include a marked increase in COVID-19 interest for *nanosilver*, *propolis*, and *antibacterial*, followed by a return to baseline levels, while *antimicrobial* maintained a sustained upward trajectory. *Biomaterials* experienced initial declines but later stabilized at elevated levels. **Conclusions**: These findings underscore the oscillating nature of public interest in antimicrobial and biomaterial innovations, highlighting opportunities for targeted research and commercialization. By adapting future material development to emerging trends and clinical needs, dentistry can use these insights to develop infection control strategies, improve restorative materials, and deal with persistent challenges such as antimicrobial resistance, peri-implantitis, and tooth caries treatment.

## 1. Introduction

The rapid emergence and global spread of multidrug-resistant microorganisms have increased the need for innovative antimicrobial solutions, particularly in fields like dentistry, where biofilm-related infections, peri-implantitis, and dental caries remain persistent challenges. Traditional antibiotics, while essential, face diminishing efficacy, prompting an urgent search for alternatives that can address the problem of increasing resistance [1,2,3]. Among these, nanosilver and propolis have emerged as promising candidates for oral infection control, wound healing, and the development of advanced dental biomaterials, including restorative composites, cement additives, and implant coatings [4,5]. Not only do these materials offer new mechanisms of antimicrobial activity, but they also align with the growing interest in sustainable and natural medical solutions [6]. Alongside traditional approaches, the public and professional focus on natural and innovative antimicrobial strategies has evolved significantly over the past decade, influenced by global health problems such as the COVID-19 pandemic [7].

Propolis, a resin-like substance produced by bees, has gained increasing attention for its strong antimicrobial and antibacterial properties, making it a valuable natural additive in healthcare and dental care applications such as mouth rinses and toothpastes [8,9]. Propolis-based gels and toothpastes have demonstrated notable effectiveness in treating periodontal disease and dental caries and supporting post-operative healing. As noted by Barboza et al., propolis shows remarkable versatility in oral therapeutic applications and has great potential as an additive to conventional dental treatment [10]. In implantology, propolis has been studied for its ability to improve osseointegration and reduce inflammation around the implant. A systematic review by Sycińska-Dziarnowska et al. highlighted its potential benefits, such as antimicrobial and tissue regenerative effects, as being valuable in reducing the risk of peri-implant inflammation and promoting soft tissue healing in implant dentistry [11]. Beyond surgical applications, propolis is also effective in preventive dentistry. Alghutaimel et al. outlined its use in mouthwashes, toothpaste, and varnishes to reduce plaque formation, inhibit cariogenic bacteria, and support overall oral hygiene [12]. Otręba et al. conducted a systematic review that further confirms the antimicrobial efficacy of propolis, particularly against pathogens responsible for oral infections, such as *Streptococcus mutans* and *Porphyromonas gingivalis*. Their findings suggest that propolis can be incorporated into therapeutic strategies in periodontal and endodontic infections [13]. Together, these studies affirm propolis as a multi-functional biomaterial with significant potential in both preventive and therapeutic dentistry. Its incorporation into various dental formulations and devices could represent a shift toward more biocompatible and naturally derived solutions for oral health management.

Similarly, nanosilver, a material known for its strong antimicrobial efficacy, has become a key player in dental material innovations, demonstrating the potential for reducing biofilm formation on implants, orthodontic appliances, and restorative surfaces. These materials represent natural and synthetic innovations, fostering new opportunities for their integration into dental biomaterial technologies with antimicrobial properties [14,15,16]. Nanosilver gained substantial attention in dentistry due to its broad-spectrum antimicrobial properties and ability to disrupt biofilms and bacterial cell walls. Their integration into dental materials has been explored across various subfields, ranging from restorative dentistry and endodontics to orthodontics and preventive care. Fernandez et al. and Mallineni et al. provided a broad overview of how nanosilver has been utilized to enhance dental materials, noting its incorporation into composites, adhesives, sealants, and implant coatings to prevent microbial colonization and improve longevity [17,18]. Wang et al. further emphasize the therapeutic advantages of silver-based biomaterials, especially for post-operative infection control, wound healing, and mucosal protection [19]. Similarly, Yin et al. discussed the growing application of antimicrobial nanoparticles-including silver-in managing caries, periodontitis, and oral candidiasis [20]. The antibacterial mechanism of nanosilver in dentistry, including their effect on bacterial membranes and intracellular functions, has been well documented with minimal resistance development and low toxicity when appropriately formulated [21]. In a follow-up review, the same research group demonstrated the promising use of silver nanomaterials in caries prevention, suggesting their role in next-generation preventive strategies [22]. From an orthodontic perspective, Sycińska-Dziarnowska et al. conducted a systematic review and meta-analysis confirming the antimicrobial efficacy of silver-enhanced bonding systems without compromising their mechanical properties, making them viable for long-term orthodontic use [23]. Additionally, another review by the same group evaluated nanosilver coatings on fixed orthodontic appliances, such as brackets, archwires, and microimplants, and reported consistent reductions in bacterial adhesion and plaque accumulation [14]. The use of nanosilver can improve clinical outcomes, reduce reliance on traditional antibiotics, and align with the broader goal of integrating advanced, evidence-based technologies into oral healthcare.

The increasing prevalence of multidrug-resistant (MDR) bacterial strains in the oral cavity presents a major challenge for contemporary dental care, particularly in the context of periodontal therapy, peri-implantitis, and post-operative infections. Silver nanoparticles have emerged as a promising alternative to traditional antibiotics due to their potent antibacterial and anti-biofilm properties, even against resistant pathogens. El-Telbany and El-Sharaki demonstrated that AgNPs exhibit strong antibacterial and anti-biofilm activity against *Pseudomonas aeruginosa* strains isolated from infected dental implants, including those with confirmed multidrug resistance. Their findings suggest that nanosilver can effectively disrupt biofilm formation and inhibit bacterial proliferation on implant surfaces, making it a valuable adjunct in peri-implant infection control [24]. Similarly, Noori Mahal et al. evaluated the efficacy of silver nanoparticles against drug-resistant strains of *Staphylococcus aureus* isolated from periodontal pockets. The study confirmed the significant bactericidal effects of nanosilver, proposing its use as a supplementary or alternative antimicrobial strategy in treating refractory periodontal infections [25]. These findings are particularly important as periodontal pathogens frequently exhibit resistance to commonly prescribed antibiotics, thereby limiting therapeutic options. Incorporation of nanosilver into dental coatings, rinses, and localized delivery systems may offer a viable strategy to reduce reliance on conventional antibiotics and manage biofilm-associated infections more effectively.

As scientific research has progressed, public awareness and curiosity about these alternatives have grown in parallel. Online search tools such as Google Trends, which analyze global search behaviors, provide a unique opportunity to assess public interest and perception over time [26]. This allows us to assess global interest and search behaviors concerning key terms. Such analysis provides valuable insights into public perceptions, knowledge gaps, and potential market trends, especially for antimicrobial strategies targeting oral health.

Google Trends offers an opportunity to assess longitudinal patterns in the public interest by analyzing search engine data. By analyzing temporal changes, this study aims to illuminate the evolving interest in questioned topics, highlight areas of public research, and suggest directions for future research and development in dentistry. Google Trends was chosen as the primary tool for this study because of its unique ability to capture real-time, large-scale patterns of public and professional interest around the world and over different time periods. As a free, publicly available platform with global reach, Google Trends provides normalized data on the number of searches, allowing researchers to assess changes in interest in specific topics over time [26]. In the context of antimicrobial biomaterials—such as propolis and nanosilver—this is particularly valuable because public awareness and professional engagement with these materials have been shaped by dynamic external events, most notably the COVID-19 pandemic. During such global health crises, Google search activity often reflects heightened concerns about infection control and alternative therapeutic options, offering a proxy for measuring information, search behavior, product interest, or emerging awareness. Furthermore, in the era of antimicrobial resistance, public curiosity, and clinical research are increasingly related, and Google Trends serves as a tool to monitor how societal concerns align with innovation in dental materials. Its application in this study enables the detection of interest surges, keyword relationships, and temporal fluctuations that would otherwise remain hidden in traditional clinical datasets. Furthermore, this analysis provides the implications of public interest for the development of innovative antimicrobial and dental biomaterial technologies.

The main objective of this study is to assess how the clinical relevance of *propolis*, *antimicrobial*, *antibacterial*, *nanosilver*, and *biomaterials* evolves over time and how these terms may shape each other’s trajectories in practice. A key goal is to determine whether external events, such as the COVID-19 pandemic, are disrupting or reinforcing these trends, thereby attempting to find out how global health crises may change both public and clinical attention to antimicrobial alternatives or dental biomaterial innovations.

## 2. Materials and Methods

To evaluate global trends in public interest regarding the terms *propolis*, *antimicrobial*, *antibacterial*, *nanosilver*, and *biomaterials*, data were collected using Google Trends (https://trends.google.com) accessed on 25 October 2024. Keywords were selected based on their relevance in current dental material literature and their cross-application across both public and professional interests. Broader terms such as *antimicrobial* and *biomaterials* were included intentionally to capture overlapping areas of interest, including both general and dentistry-specific innovation. Google Trends is a web-based tool that provides access to the relative frequency of search terms entered into the Google search engine. The methodology for the data collection and analysis is outlined below.

### 2.1. Search Parameters

The search terms used were *propolis*, *antimicrobial*, *antibacterial*, *nanosilver*, and *biomaterials*. The scope of the analysis was set to “Worldwide” to capture global search patterns. Data were retrieved for the last ten years (spanning from November 2014 to the end of October 2024). The general category was used to avoid limiting the search to specific fields (e.g., healthcare or materials science).

### 2.2. Data Extraction

Relative search interest for each term was retrieved and reported as a normalized value ranging from 0 to 100, where 100 represents the highest search frequency for a term during the selected period. Trends were visualized as time series data to identify patterns of interest over the ten-year period.

### 2.3. Data Processing

Extracted data were exported as CSV files from Google Trends for further analysis.

Data were inspected for missing values using summary statistics and visual plots, and none were found. The data were then differenced to achieve stationarity, as confirmed by the Augmented Dickey–Fuller (ADF) test. No additional transformations (e.g., logarithmic) were applied, as the differenced data were approximately normally distributed. By analyzing longitudinal Google Trends data over the period 2014–2024 through multivariate time series methods, including vector autoregressive (VAR) modeling and Impulse Response Function (IRF) analysis, the investigation aims to quantify the extent to which these topics are interdependent, offering insights into emerging or diminishing interests that could inform evidence-based innovative device coatings and integrative strategies for infection control.

### 2.4. Statistical Analysis

All statistical analyses were conducted with a two-sided alpha level set at α = 0.05. The distribution of the numeric variables was reported using median means (*M*), standard deviation (*SD*), medians (*Mdn*), and interquartile ranges (*IQR*).

The stationarity of the time series was examined using the Augmented Dickey–Fuller Test with four lags. As a result of the stationarity analysis, further analysis was performed on the first differences of the values Δ*y_t_*, which were calculated using the following formula:

Δ*y_t_* = *y_t_* − *y_t_*_−1_ (1)

where *y_t_* is the value of the series at time *t*, and *y_t_*_−1_ is the value of the series at the previous time step.

The data fitting was conducted using vector autoregression (VAR) models with a lag order of (*p* = 5), selected based on model fit and information criteria. We evaluated lag orders from (*p* = 1) to (*p* = 8). The model with (*p* = 5) was chosen because it maximized the adjusted R^2^ and overall R^2^ compared to shorter lag orders (e.g., (*p* = 3, 4)), indicating a better balance between explanatory power and complexity (Table S1). Further increasing the lag order (e.g., (*p* = 6, 7, 8)) did not significantly improve adjusted R^2^ and risked overfitting due to additional parameters. The Akaike Information Criterion (AIC) is also supported (*p* = 5) as an optimal lag order for most keyword combinations. Sensitivity analyses with alternative lag orders confirmed the robustness of the primary findings.

The data fitting was conducted using vector autoregression (VAR) models with a lag order of *p* = 5 and a deterministic trend regression using the equation. Ordinary Least Squares (OLS) estimation was applied separately to each equation of the system.

*y_t_* = *c* + Φ_1_·*y_t_*_−1_ + Φ_2_·*y_t_*_−2_ + Φ_3_·*y_t_*_−3_ + Φ_4_·*y_t_*_−4_ + Φ_5_·*y_t_*_−5_ + *γ_t_* + *u_t_* (2)

where *y* denotes the k-dimensional vector of endogenous variables at time *t*; *c* denotes the k-dimensional vector of intercept terms; Φ_1_, Φ_2_, Φ_3_, Φ_4_, and Φ_5_ denote *K* × *K* coefficient matrices capturing the relationships between lags of the endogenous variables; *γ_t_* denotes the deterministic linear trend (vector of trend coefficients); *u_t_* denotes the *K*-dimensional vector of error terms, assumed to be white noise with zero mean and constant variance; and *K* denotes the number of the endogenous variables.

The *VAR*(*p*) process was confirmed as stable by meeting the condition that eigenvalues of the companion matrix *A* have modulus less than 1.

*Det* (*I_K_* − *A*_1__*z*_ − …*Ap·zp*) ≠ 0 *for |z| ≤* 1 (3)

where *Det* denotes the determinant operation, *I_K_* denotes the *K* × *K* identity matrix, *Ai* (*i* = *1*, *2*,…, *p*): *K* × *K* denotes coefficient matrices corresponding to the lag *i* in the *VAR*(*p*) process, and *z* denotes a complex number used in the polynomial.

All models were tested for changing levels of variability over time using the Autoregressive Conditional Heteroscedasticity Lagrange Multiplier (ARCH-LM) test. To ensure no lingering correlation in the model’s residuals up to 20 time periods, we applied the multivariate asymptotic Portmanteau test. We also checked for both immediate (instant) and delayed (lagged) cause–effect relationships among the keywords. The instant relationships were identified using a Wald-type test, while delayed causality was assessed with the F-type Granger causality test.

To confirm that our vector autoregressive (VAR) model was stable across the observation period, an empirical fluctuation process using the Ordinary Least Squares (OLS) cumulative sum (CUSUM) method was calculated. Next, it was estimated how each keyword’s popularity would respond over time to a sudden one-unit increase (or “shock”) in another keyword. These responses were represented by impulse response coefficients (IRF) and included 95% confidence intervals derived by a bootstrap approach with 100 replications. Finally, to determine which keyword contributed the most to future fluctuations in the others, we performed forecast error variance decomposition (FEVD), allowing us to see how much of each keyword’s predicted change can be attributed to shifts in the other keywords.

#### 2.4.1. Characteristics of the Statistical Tool

Analyses were conducted using the R Statistical language (version 4.3.3; R Core Team) [27] on Windows 11 x64 (build 22631) and the package *report* (version 0.5.8; Makowski) [28].

#### 2.4.2. Characteristics of the Study Sample

**Figure 1:**
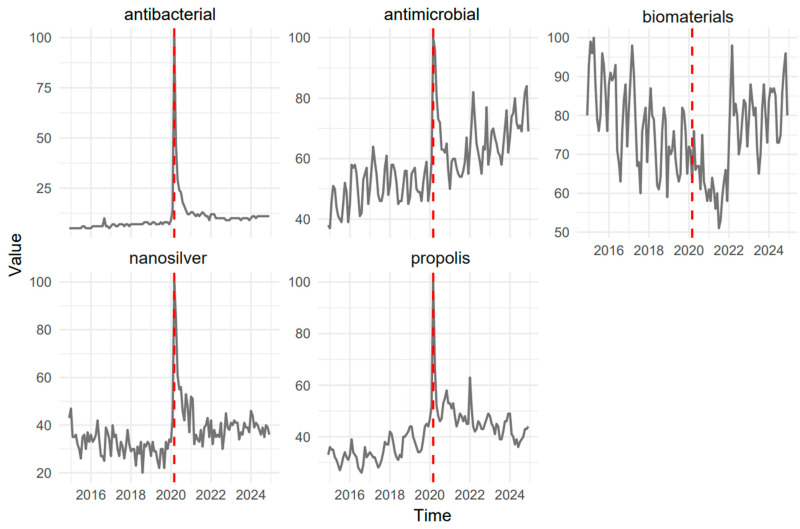
Distribution of time-based interest of keywords. The dashed red line marks the beginning of the COVID-19 pandemic (March 2020).

An evaluation of Google Trends data was performed to examine monthly search interest in *propolis*, *antimicrobial*, *antibacterial*, *nanosilver*, and *biomaterials* from November 2014 to October 2024 (120 observations per term). Half of these data points correspond to the pre-COVID-19 period (November 2014–early 2020) and half to the COVID-19 era (March 2020–2023). The COVID-19 era was defined as starting in March 2020, corresponding to the World Health Organization’s declaration of COVID-19 as a global pandemic on 11 March 2020, which marked a global surge in health-related search interest. Each query’s interest score was standardized on a 1–100 scale by Google, enabling direct comparisons across keywords (Figure 1).

The visualization of the time series in Figure 1 shows that *antibacterial*, *nanosilver*, and *propolis* experienced marked surges at the onset of COVID-19 (March 2020), followed by transient declines showing a shift in public and professional engagement with these topics in relation to pandemic timing. In contrast, *antimicrobial* exhibited a more sustained upward trajectory across the entire observation window, with further amplification during the pandemic. *Biomaterials* displayed a downward trend from 2014 to 2021, then reached a localized peak in early 2022, ultimately stabilizing at an elevated level.

Notably, *nanosilver* showed a gradual decrease in interest prior to COVID-19, which was reversed by a pronounced spike during early 2020, thereafter consolidating at a slightly higher baseline (see Table 1 with descriptive statistics). *Antibacterial* remained consistently low before the pandemic, underwent an abrupt surge at its onset, and then settled to marginally heightened levels. *Propolis* demonstrated moderate pre-COVID-19 fluctuation but experienced a substantial rise during the initial pandemic phase, followed by a gradual return to higher-than-baseline values.

## 3. Results

The results of the Augmented Dickey–Fuller test for raw keyword scores and for the differences are presented in Table 2. The results of the fitted VAR (*p* = 5) model, with the matrix of keyword interest levels as endogenous variables and the period factor (baseline coded as 0 and COVID-19 surge coded as 1) as an exogenous factor, are presented in Tables S2–S6 in Supplementary Materials.

### 3.1. Validation and Diagnostics of the VAR Model

The obtained eigenvalues, ranging from 0.18 to 0.894, confirmed the stability of the VAR model. Additionally, the model was conditionally homoskedastic, as indicated by the ARCH-LM test χ^2^(1125) = 1144.9, *p* = 0.333, and exhibited no evidence of serial correlation, as shown by the Portmanteau test χ^2^(375) = 389.09, *p* = 0.297.

**Figure 2:**
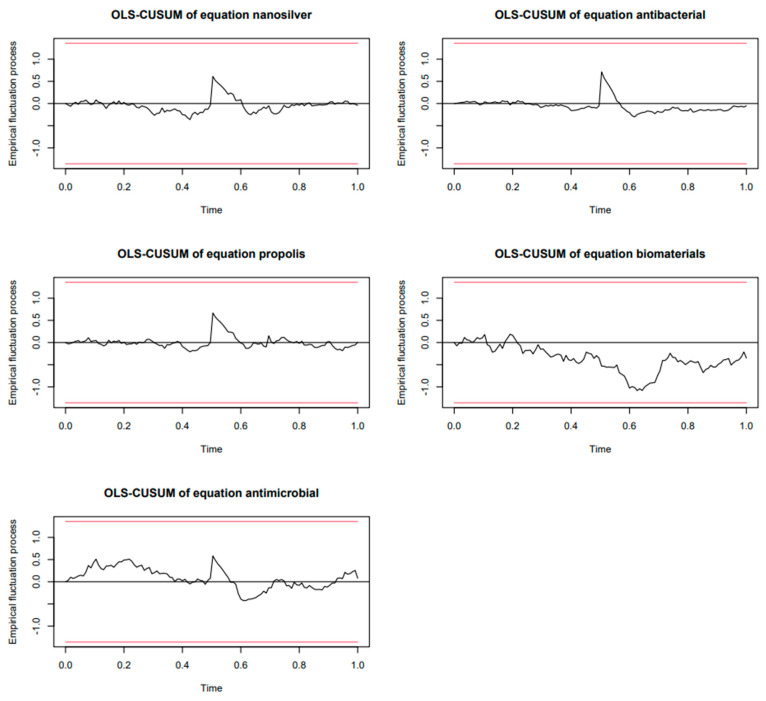
OLS-CUSUM analysis of population stability for studied healthcare keywords over 2014–2024.

The OLS-CUSUM analysis in Figure 2 showed stability patterns varying among the keywords. Thus, *nanosilver* exhibited a highly stable trend, with minimal variability and a flat trajectory throughout the timeline, except for the COVID-19 surge. *Biomaterials* displayed moderate fluctuations, but these variations also stayed well within the confidence bounds. *Antibacterial* and *propolis* demonstrated short-lived deviations during the COVID-19 surge, momentarily increasing in variability but never crossing the red thresholds, signifying that the changes were transient and statistically insignificant. *Antimicrobial* showed more pronounced and sustained fluctuations compared to the other keywords, particularly around and after the COVID-19 surge; however, even these fluctuations remained confined within the stability boundaries. The overall impact of COVID-19 on the stability of interest in the analyzed keywords was generally noticeable but relatively short-term, lasting no more than six months for each of the studied keywords.

The analysis of causality effects in Table S7 for the fitted VAR model demonstrates clear distinctions in how keyword popularity influences other keywords, both instantaneously and with delays. Instantaneous causality results showed strong and statistically significant effects (*p* < 0.001) for all keywords, demonstrating that changes in the popularity of any one keyword are immediately associated with changes in others. This indicates strong interdependencies between the keywords in real time.

Delayed causality results revealed more nuanced patterns. *Antibacterial* and *biomaterials* exhibited significant delayed causality effects (*p* < 0.001), indicating that fluctuations in these keywords have a measurable impact on others over time. In contrast, *nanosilver* demonstrated weaker but statistically significant delayed effects (*p* = 0.042), showing a less pronounced but still detectable influence over time. *Propolis* and *antimicrobial*, however, did not show statistically significant delayed causality (*p* = 0.215 and *p* = 0.151, respectively), implying that their impacts are primarily instantaneous and do not persist significantly into later periods.

### 3.2. Analysis and Interpretation of the Fitted VAR Model Results

Across the five VAR models, a broad pattern emerges of each keyword’s short-term momentum, often moderating itself at the next lag and hinting at saturation effects in digital attention. For the *nanosilver* (Table S2) keyword, its own lagged values show robust negative coefficients (e.g., β = −0.66, *SE* = 0.19, *p* = 0.001 at lag 1), implying that an immediate upswing in *nanosilver*-related interest can taper off in the subsequent period.

A similar autocorrecting phenomenon appears for *antibacterial* when modeled as the outcome (Table S5): once *antibacterial* experiences an elevated level (lag 1), it tends to recede in the next step (β = −0.67, *SE* = 0.27, *p* = 0.016).

By contrast, *antimicrobial* as the dependent variable (Table S4) exhibits a noteworthy overall fit (F(27, 88) = 3.68, *p* < 0.001; *R*^2^ = 0.53), underlining the relatively intricate network of influences acting on it. *Antimicrobial* has a strongly negative autoregressive term at lag 1 (β = −0.87, *SE* = 0.19, *p* < 0.001), accompanied by significant positive impacts from *propolis* and *nanosilver* in later lags (e.g., *propolis* at lag 1: β = 0.53, *SE* = 0.20, *p* = 0.008; *nanosilver* at lag 5: β = 0.24, *SE* = 0.12, *p* = 0.044). These estimates reflect that an initial surge in *antimicrobial* interest is often followed by a downshift, whereas rising searches for *propolis* or *nanosilver* can prime future increases in *antimicrobial* queries.

**Figure 3:**
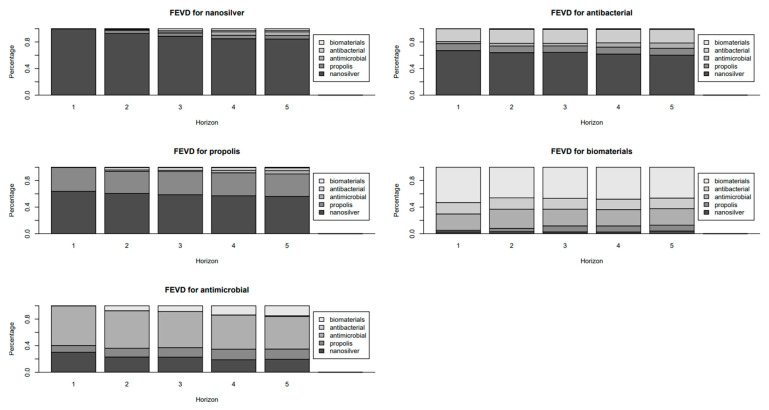
Results of the forecast error variance decomposition for individual keywords.

The results of the forecast error variance decomposition for individual keywords are shown in Figure 3.

*Propolis* itself (Table S3) is shaped by *antibacterial* fluctuations at lag 1 (β = −0.38, *SE* = 0.18, *p* = 0.041): elevated *antibacterial* searches appear to drain short-term interest in *propolis*, perhaps illustrating how users pivot from one therapeutic angle to another.

Meanwhile, the *biomaterials* query (Table S6) shows meaningful relationships with *nanosilver* at lags 4 and 5 (e.g., lag 5: β = 0.42, *SE* = 0.15, *p* = 0.005). These results indicate that a pronounced *nanosilver* presence earlier on can later spur additional exploration of *biomaterials*. Nonetheless, *biomaterials* also exhibit a negative coefficient for *antimicrobial* at lag 1 (β = −0.55, *SE* = 0.24, *p* = 0.023) and a pronounced self-lag effect at lag 3 (β = −0.52, *SE* = 0.18, *p* = 0.004), reflecting how a burst of interest in *antimicrobial* may momentarily redirect attention away from *biomaterials*, and how *biomaterials* itself can revert downward after a prior rise. The above results illustrate that *nanosilver*, *propolis*, *antimicrobial*, *antibacterial*, and *biomaterials* each manifest both independent trajectories and meaningful cross-influences over time.

### 3.3. Analysis and Interpretation of Impulse Response Function (IRF) Results

The results of impulse response analyses of Google Trends data (2014–2024) in Table S8 demonstrate that surges in public interest for one keyword can drive immediate or short-term changes in related topics, as evidenced by statistically significant coefficients (i.e., 95% confidence intervals not crossing zero).

For instance, an immediate rise in *nanosilver* searches corresponds to higher interest in *propolis* (coefficient = 5.03, *95% CI*: 1.29–7.96), *antimicrobial* (3.22, *95% CI*: 0.57–4.60), and *antibacterial* (7.67, *95% CI*: 1.49–12.51), stressing nanosilver’s recognized antimicrobial properties and its capacity to stimulate parallel explorations of other natural or antibacterial agents.

In contrast, an increase in *propolis* queries about propolis has a positive lag-one effect on interest in *nanosilver* (1.89, *95% CI*: 0.41–3.75), suggesting that once people explore *propolis*, they subsequently look for *nanosilver.*

*Propolis* also shows notable immediate effects on *antimicrobial* (1.87, *95% CI*: 0.53–2.47) and *antibacterial* (3.03, *95% CI*: 1.03–3.64) keywords, reinforcing the idea that individuals investigating propolis (a traditional natural therapy) often branch into broader antibacterial approaches at the same time. Meanwhile, *antimicrobial* interest drives a significant positive immediate effect on *antibacterial* (1.62, *95% CI*: 0.50–2.04). In addition, *antimicrobial* searches produce a positive effect on *biomaterials* at lag 0 (3.62, *95% CI*: 1.61–4.46) but a negative effect at lag 1 (−2.17, *95% CI*: −3.05–−0.73).

Finally, biomaterials to antimicrobial displays a similar pattern: after a one-step lag, an increase in *biomaterials* queries significantly lowers subsequent *antimicrobial* interest (−1.98, *95% CI*: −2.58–−0.79), which may reflect the public or clinical audience pivoting away from broad antimicrobial strategies once they encounter specifics about novel biomaterials. Clinically, these findings stress the short-lived but meaningful ripple effects that attention to one therapeutic avenue can have on related domains, particularly at early lags, demonstrating the need for timely, integrated clinical information when novel or complementary treatments (e.g., *nanosilver*, *propolis*, *or other biomaterials*) rise in popularity.

### 3.4. Analysis and Interpretation of Forecast Error Variance Decomposition (FEVD) Results

The phenomenon of how fluctuations in one keyword can explain future variability in the others over a five-step horizon by applying forecast error variance decompositions on the fitted VAR model visualized in Figure 3 and summarized in Table S9 illustrates how the popularity of a keyword in one domain influences variability in related domains, offering insights into public and clinical interest in antimicrobial and biomaterial solutions. These findings align with the notion that the population of a keyword in one domain (e.g., *nanosilver*) can ripple into inquiries on complementary areas (e.g., *propolis*, *antibacterial*) and that these relationships evolve over time.

*Nanosilver*

At step 1, nanosilver’s forecast error variance is entirely self-explained (100%), indicating that initial fluctuations are driven by their own dynamics. By step 5, approximately 15.52% of its variance is attributed to other keywords: propolis (5.21%), antimicrobial (5.59%), antibacterial (2.14%), and biomaterials (2.57%) (Table S9). This indicates that while nanosilver retains a strong independent trajectory, related keywords contribute modestly to its variability over time, likely reflecting shared interest in antimicrobial applications. This underscores nanosilver’s role as a focal point for broader inquiries into alternative antimicrobial agents.

*Propolis*

Propolis shows significant dependence on nanosilver, which explains 56.07% of its variance by step 5, up from 63.76% at step 1 (Table S9). Propolis itself accounts for 33.78% of its variance at step 5, with antimicrobial (5.03%), antibacterial (4.25%), and biomaterials (0.87%) contributing minimally. This strong nanosilver influence indicates that surges in nanosilver interest, often tied to its antimicrobial properties, drive subsequent propolis searches, a natural antimicrobial adjunct. This aligns with the IRF results (Table S8), where nanosilver shocks positively impact propolis at lag 0 (coefficient = 5.03, *95% CI*: 1.29–7.96). These patterns imply that from both clinical and patient perspectives, exploring nanosilver-based solutions frequently investigates propolis as a complementary therapy.

*Antimicrobial*

At step 1, antimicrobial explains 59.74% of its own variance, with nanosilver (30.08%) and propolis (10.18%) as notable contributors. By step 5, its self-explanation decreases to 48.86%, with nanosilver (19.50%), propolis (15.43%), biomaterials (14.97%), and antibacterial (1.24%) accounting for the remainder (Table S9). This indicates that broad antimicrobial interest is increasingly shaped by specific inquiries into nanosilver and propolis, reflecting their prominence in discussions about antibiotic-resistant pathogens. The IRF results support this, showing significant lagged effects from propolis (lag 1: coefficient = 1.59, *95% CI*: 0.25–2.54) and nanosilver (lag 5: coefficient = 0.24, *95% CI*: 0.01–0.47). Clinically, this demonstrates that novel antimicrobial agents sustain engagement with the broader antimicrobial category.

*Antibacterial*

Antibacterial’s variance is heavily influenced by nanosilver, which explains 60.10% by step 5, compared to 67.06% at step 1 (Table S9). Antibacterial itself accounts for 20.19% at step 5, with propolis (10.44%), antimicrobial (7.97%), and biomaterials (1.30%) contributing less. This dominant nanosilver influence highlights its role in catalyzing targeted antibacterial inquiries, likely due to its efficacy against resistant bacteria. The IRF results corroborate this, showing a significant immediate effect of nanosilver on antibacterial interest (coefficient = 7.67, *95% CI*: 1.49–12.51). These dynamics reveal that nanosilver serves as a gateway to specific antibacterial searches, informing infection control strategies.

*Biomaterials*

Biomaterials remain largely self-driven, explaining 46.39% of its variance at step 5, down from 53.19% at step 1 (Table S9). Antimicrobial contributes significantly (24.79%), followed by antibacterial (15.98%), propolis (8.85%), and nanosilver (4.00%). This relative independence reveals that biomaterials maintain a distinct trajectory driven by specialized research in advanced material applications. The IRF results align, showing a significant but short-lived antimicrobial effect on biomaterials at lag 0 (coefficient = 3.62, *95% CI*: 1.61–4.46), followed by a negative effect at lag 1 (coefficient = −2.17, *95% CI*: −3.05–−0.73). Clinically, this indicates that while antimicrobial inquiries briefly spur biomaterial interest, the latter’s trajectory is primarily driven by internal dynamics, such as innovations in device coatings.

*Clinical and research implications*

The FEVD results highlight the interconnected nature of public and clinical interest in these keywords. Nanosilver’s strong influence on propolis and antibacterial indicates that emerging antimicrobial agents drive engagement with related therapies, offering opportunities for integrative wound care protocols. The significant self-explanation of biomaterials underscores the need for targeted research to bridge general antimicrobial interest with specialized applications. These findings, combined with IRF results, provide a comprehensive view of keyword interdependencies, guiding clinicians and researchers in leveraging online interest for practical interventions.

## 4. Discussion

The analysis of Google Trends data from the past decade reveals significant insights into global public interest in the topics of *propolis*, *antimicrobial*, *antibacterial*, *nanosilver*, and *biomaterials*—all of which play increasing importance in the field of dentistry. These terms represent the convergence of traditional natural remedies and cutting-edge material science, both of which are increasingly important due to the ongoing challenges posed by antimicrobial resistance and the need for advanced biomaterials in healthcare and dentistry. By leveraging Google Trends data and statistical analyses, it has provided valuable insights into the temporal and contextual factors influencing these interests, offering implications for dental research, clinical practices, and biomaterial development. The findings of this study, while grounded in statistical modeling, carry meaningful implications for dental research, innovation, and clinical practice. The observed surges in public interest for terms like *nanosilver* and *propolis*, particularly during the COVID-19 period, reflect a growing curiosity about alternative antimicrobial strategies, many of which are directly relevant to dental applications. Nanosilver, for instance, is increasingly incorporated into dental materials such as orthodontic brackets, implant coatings, and restorative composites due to its broad-spectrum antimicrobial properties [5,14,23]. Similarly, propolis, which is valued for its natural origin and biocompatibility, has been explored in mouthwashes, toothpaste, periodontal therapy, and oral wound healing [11,12,13]. The correlations and impulse responses between these terms suggest that patient or consumer interest in one antimicrobial agent can trigger curiosity in others, potentially influencing product demand, patient inquiries, or future market trends. By aligning dental material development and patient education with these emerging patterns of interest, the dental field can better respond to evolving expectations, particularly around infection prevention and the use of natural or nanomaterial-based agents in oral care.

The analysis reveals a consistent increase in public interest in *nanosilver*, *propolis*, and related terms over the study period, reflecting a growing awareness of antimicrobial resistance and the need for alternative solutions in dentistry. It is noteworthy that the increase in search activity coincided with the COVID-19 pandemic, suggesting that global health crises can significantly amplify public interest in antimicrobial and antibacterial strategies [3,29]. This finding underscores the importance of public health campaigns and educational initiatives that can harness such moments to promote informed decision-making and evidence-based use of alternative antimicrobial products [30].

The prominence of *propolis* in search trends may be attributed to its natural origin and widespread perception as a safe, sustainable alternative to synthetic antimicrobials [4,31]. This is consistent with the growing interest in natural additives for periodontal therapy, mouthwashes, and oral hygiene products. Similarly, the continued interest in nanosilver reflects its multipurpose and well-established role in dental applications, including antimicrobial coatings for implants, orthodontic brackets, and tooth restoration materials [14,23,32,33]. The intersection of these interests highlights a potential avenue for combining natural and nanomaterial-based antimicrobials in future research and dental product development. This study’s results reinforce the relevance of nanosilver and propolis in addressing key challenges in dental infection control and biomaterial development. Nanosilver, with its broad-spectrum antimicrobial properties and demonstrated efficacy against antibiotic-resistant pathogens, continues to be a cornerstone of biomedical and dental innovation [34]. However, concerns surrounding nanosilver’s potential cytotoxicity and environmental impact warrant ongoing investigation to optimize its safety and efficacy in clinical applications, particularly in dental pulp capping, endodontic sealers, and periodontal treatments [17,18,35,36].

On the other hand, propolis offers a natural and biocompatible alternative, particularly in wound healing in oral surgery, periodontal therapy, and mucosal injuries. Its rich composition of bioactive compounds, including flavonoids and phenolics, underpins its antimicrobial activity and positions it as a valuable adjunct in the treatment of infections [11,12,37,38]. Future research should explore the synergistic potential of combining propolis with nanosilver or other biomaterials to enhance their joint efficacy while mitigating potential limitations. The overlap between search terms such as antimicrobial and nanosilver suggests a growing awareness of the intersection between antimicrobial technologies and advanced materials. However, the relatively distinct trends for propolis may indicate its positioning more as a traditional or alternative therapeutic agent than as a major biomaterial despite its demonstrated antimicrobial properties. This delineation presents an opportunity for further integration of natural substances such as propolis into modern dental biomaterial science, particularly in areas like caries prevention, periodontal therapy, and implant coatings.

The COVID-19 pandemic had no impact on antimicrobial resistance but raised awareness of preventive measures [29,39]. This phenomenon reflects the increased awareness of infection prevention during global health crises and demonstrates how such events can shape both public behavior and research priorities [40]. For example, the increased emphasis on hand sanitizers, disinfectants, and antimicrobial surfaces during the pandemic may have indirectly fueled interest in nanosilver [41,42]. The COVID-19 pandemic has further highlighted the importance of alternative antimicrobial strategies, providing a timely reminder of the need to invest in dental research, education, and innovation. In addition, despite initial assumptions that the COVID-19 pandemic might have reshaped these patterns, the “COVID surge” variable never attains statistical significance (*p*-values > 0.05 for all keywords). Consequently, no substantial realignment appears in the studied timelines, indicating that clinical and public interest in alternative antimicrobials or biomaterials followed internal dynamics rather than permanently redirected by the pandemic. The estimated outcomes indicate that interest in novel modalities, such as *nanosilver* or *propolis*, may oscillate alongside interest in more conventional categories, such as *antibacterial* and *antimicrobial*, with shifts tending to be self-correcting yet capable of influencing adjacent areas of dental relevance.

While Google Trends offers a unique lens into public interest, its limitations should be acknowledged. The relative nature of the data does not reflect absolute search volumes, and variations in internet accessibility and cultural differences may bias results. Additionally, the tool does not capture the depth or sentiment of the queries, which would require supplementary qualitative analysis. The precise structure of Google’s algorithms remains undisclosed by the company. Inconsistencies in phrasing or methodological errors in analyzing queries from a single Google search user may be inherent limitations of such research. Various factors can influence Google search queries, and the potential impact of external elements cannot be entirely eliminated [43]. Phrase overlaps or inconsistencies in how a single user formulates search queries are inherent limitations of studies based on Google search data. Various factors can influence search behavior, and the impact of external influences on the results cannot be entirely eliminated. Search data cannot differentiate between public, commercial, or professional dental interests. There is no regional or language-level granularity, potentially overlooking local variations in dental material use or public awareness

Future research should explore the integration of Google Trends data with other sources, such as social media analytics, to create a more comprehensive understanding of public engagement with these topics. Furthermore, longitudinal studies examining the correlation between search trends and actual scientific advancements or health outcomes could provide deeper insights into the reciprocal relationship between public interest and dental innovation.

## 5. Conclusions

This study shows that public interest in antimicrobial agents and biomaterials, particularly nanosilver and propolis, has fluctuated over the past decade in patterns that reflect global health dynamics, public curiosity, and emerging scientific topics. While a visible spike in search activity was observed during the early phase of the COVID-19 pandemic, statistical modeling revealed that the “COVID surge” variable did not consistently show significant long-term effects across all search terms. Therefore, the pandemic can be interpreted as a temporary amplifier of interest rather than the ultimate driver of lasting change.

Importantly, the findings reveal clear interdependencies between search terms, with nanosilver and propolis and queries related to antimicrobial and antibacterial agents, highlighting their relevance as complementary materials in oral infection control. These dynamics suggest that increased public attention to natural and nanomaterial-based antimicrobials can be used to guide innovation in dental materials, such as antimicrobial composites, implant coatings, and biocompatible surface modifiers.

Rather than offering definitive conclusions about clinical adoption, this study presents search behavior as a proxy for awareness, signaling opportunities for dental researchers and manufacturers to align material development with evolving public interest. By monitoring these digital patterns, the dental field can better anticipate patient expectations, inform product design, and prioritize areas of research that resonate with broader health concerns, such as antimicrobial resistance and biocompatibility.

## Figures and Tables

**Table 1:** Descriptive statistics of the level of interest [1–100] in keywords based on the time period 11.2014–10.2024.

Keyword	n	Period	M	SD	Mdn	IQR
Nanosilver	60	Baseline	31.61	4.96	32.00	29.00–35.00
	60	COVID-19 surge	41.85	11.43	39.00	36.00–42.50
Propolis	60	Baseline	34.72	4.84	34.00	32.00–38.00
	60	COVID-19 surge	47.20	9.05	46.00	43.00–49.00
Antimicrobial	60	Baseline	50.48	6.25	50.00	46.00–56.00
	60	COVID-19 surge	66.58	10.11	64.60	59.00–72.00
Antibacterial	60	Baseline	6.53	1.08	7.00	6.00–7.00
	60	COVID-19 surge	13.82	12.71	11.00	10.00–12.00
Biomaterials	60	Baseline	78.48	11.00	79.00	69.75–88.00
	60	COVID-19 surge	73.63	11.28	73.00	63.75–83.00

**Table 2:** Results of the Augmented Dickey–Fuller test for raw keyword scores and for differences.

Keyword	Type of Score	Dikey-Fuller Statistic	Lag Order	*p*
Nanosilver	Raw	−3.54	4	**0.042**
	First difference	−6.21	4	**<0.01**
Propolis	Raw	−2.74	4	0.268
	First difference	−6.82	4	**<0.01**
Antimicrobial	Raw	−4.08	4	**<0.01**
	First difference	−7.08	4	**<0.01**
Antibacterial	Raw	−3.58	4	**0.038**
	First difference	−6.82	4	**<0.01**
Biomaterials	Raw	−2.76	4	0.260
	First difference	−7.76	4	**<0.01**

## Data Availability

The original contributions presented in this study are included in the article and Supplementary Materials. Further inquiries can be directed to the corresponding author.

## Supplementary Materials

The following supporting information can be downloaded at https://www.mdpi.com/article/10.3390/dj13060253/s1, Tables S1–S9. Table S1. Lag order selection metrics for VAR model. Table S2. Results of the VAR model with *nanosilver* difference as the outcome. Table S3. Results of the VAR model with *propolis* difference as the outcome. Table S4. Results of the VAR model with *antimicrobial* difference as the outcome. Table S5. Results of the VAR model with *antibacterial* difference as the outcome. Table S6. Results of the VAR model with *biomaterials* difference as the outcome. Table S7. Results of the causality effects of keyword popularity on other keywords using instantaneous and delayed approaches. Table S8. IRF coefficients with *95% CI* according to the impulse and response keywords. Table S9. Forecast Error Variance Decomposition (FEVD) contributions at steps 1, 3, and 5.
