# Supplementary material for: Exploring Global Interest in Propolis, Nanosilver, and Biomaterials: Insights and Implications for Dentistry from Big Data Analytics
Source: Dent J (Basel). 2025 Jun 6;13(6):253. doi: 10.3390/dj13060253 (PMC12192092; doi:10.3390/dj13060253)
Supplement: Supplementary file 1 [file dentistry-13-00253-s001.zip › dentistry-3549715-supplementary.pdf]

**Supplementary materials Table S1-S9**

**Table S1.** Lag order selection metrics for VAR model.

| *Lag Order* | *$R^2$ (Average)* | *$R^2$adj (Average)* | *AIC* |
|---|---|---|---|
| 1 | 0.15 | 0.05 | 12.5 |
| 2 | 0.20 | 0.08 | 12.3 |
| 3 | 0.25 | 0.10 | 12.1 |
| 4 | 0.28 | 0.12 | 11.9 |
| **5** | **0.30** | **0.15** | **11.8** |
| 6 | 0.31 | 0.14 | 11.9 |
| 7 | 0.32 | 0.13 | 12.0 |
| 8 | 0.33 | 0.12 | 12.1 |

*Note*: The lag order ($p = 5$) was selected based on the highest ($R^2$adj) and lowest AIC values, indicating optimal model fit and parsimony.

**Table S2.** Results of the VAR model with *nanosilver* difference as the outcome.

| *terms* | *Estimate* | *SE* | *t* | *Pr(>\|t\|)* |
|---|---|---|---|---|
| trend | 0.00 | 0.03 | -0.05 | 0.961 |
| time period [COVID surge vs. baseline (ref.)] | 0.16 | 2.65 | 0.06 | 0.952 |
| | *Lag 1* | | | |
| nanosilver | -0.66 | 0.19 | -3.39 | **0.001** |
| propolis | 0.24 | 0.27 | 0.89 | 0.377 |
| antimicrobial | 0.02 | 0.26 | 0.07 | 0.941 |
| antibacterial | 0.27 | 0.24 | 1.14 | 0.259 |
| biomaterials | 0.12 | 0.15 | 0.79 | 0.434 |
| | *Lag 2* | | | |
| nanosilver | -0.47 | 0.24 | -2.01 | **0.048** |
| propolis | 0.20 | 0.26 | 0.76 | 0.450 |
| antimicrobial | 0.26 | 0.29 | 0.90 | 0.370 |
| antibacterial | -0.04 | 0.30 | -0.13 | 0.895 |
| biomaterials | -0.11 | 0.20 | -0.55 | 0.587 |
| | *Lag 3* | | | |
| nanosilver | -0.18 | 0.24 | -0.76 | 0.451 |
| propolis | 0.24 | 0.26 | 0.94 | 0.352 |
| antimicrobial | -0.08 | 0.28 | -0.29 | 0.773 |
| antibacterial | -0.10 | 0.32 | -0.30 | 0.762 |
| biomaterials | -0.19 | 0.20 | -0.99 | 0.324 |
| | *Lag 4* | | | |
| nanosilver | -0.05 | 0.20 | -0.22 | 0.825 |
| propolis | 0.36 | 0.25 | 1.43 | 0.157 |
| antimicrobial | 0.03 | 0.26 | 0.11 | 0.913 |
| antibacterial | -0.17 | 0.28 | -0.59 | 0.554 |
| biomaterials | -0.02 | 0.20 | -0.11 | 0.911 |
| | *Lag 5* | | | |
| nanosilver | -0.02 | 0.16 | -0.11 | 0.912 |
| propolis | 0.18 | 0.26 | 0.68 | 0.498 |
| antimicrobial | -0.15 | 0.20 | -0.76 | 0.452 |
| antibacterial | -0.08 | 0.22 | -0.35 | 0.730 |
| biomaterials | 0.05 | 0.17 | 0.28 | 0.784 |
| $N_{obs}$ | 115 | | | |
| $F$ test | (27, 88) = 1.45, $p$ = 0.152 | | | |
| $R^2/R^2_{adj}$ | 0.29/ 0.07 | | | |

*Note: Estimate* – the regression coefficient; *SE* – standard error; *t* – statistic of the test t; *Pr(>|t|)* – p -value of the statistical test.

**Table S3.** Results of the VAR model with *propolis* difference as the outcome.

| *terms* | *Estimate* | *SE* | *t* | *Pr(>\|t\|)* |
|---|---|---|---|---|
| trend | 0.02 | 0.02 | 0.76 | 0.450 |
| time period [COVID surge vs. baseline (ref.)] | -1.39 | 2.07 | -0.67 | 0.503 |
| *Lag 1* | | | | |
| nanosilver | 0.11 | 0.15 | 0.71 | 0.479 |
| propolis | 0.04 | 0.21 | 0.19 | 0.853 |
| antimicrobial | 0.00 | 0.21 | 0.02 | 0.987 |
| antibacterial | -0.38 | 0.18 | -2.07 | **0.041** |
| biomaterials | -0.09 | 0.12 | -0.76 | 0.447 |
| *Lag 2* | | | | |
| nanosilver | 0.24 | 0.18 | 1.28 | 0.203 |
| propolis | -0.18 | 0.21 | -0.88 | 0.379 |
| antimicrobial | 0.04 | 0.23 | 0.18 | 0.854 |
| antibacterial | -0.40 | 0.24 | -1.69 | *0.094* |
| biomaterials | -0.06 | 0.15 | -0.41 | 0.685 |
| *Lag 3* | | | | |
| nanosilver | 0.25 | 0.19 | 1.35 | 0.182 |
| propolis | -0.06 | 0.20 | -0.27 | 0.785 |
| antimicrobial | -0.20 | 0.22 | -0.91 | 0.364 |
| antibacterial | -0.35 | 0.25 | -1.44 | 0.155 |
| biomaterials | -0.10 | 0.15 | -0.65 | 0.518 |
| *Lag 4* | | | | |
| nanosilver | 0.10 | 0.16 | 0.65 | 0.517 |
| propolis | 0.00 | 0.20 | 0.02 | 0.987 |
| antimicrobial | -0.02 | 0.20 | -0.08 | 0.940 |
| antibacterial | -0.28 | 0.22 | -1.26 | 0.212 |
| biomaterials | -0.07 | 0.15 | -0.48 | 0.634 |
| *Lag 5* | | | | |
| nanosilver | 0.10 | 0.13 | 0.78 | 0.437 |
| propolis | 0.13 | 0.20 | 0.62 | 0.535 |
| antimicrobial | -0.24 | 0.16 | -1.52 | 0.132 |
| antibacterial | -0.16 | 0.17 | -0.93 | 0.357 |
| biomaterials | 0.02 | 0.14 | 0.18 | 0.861 |
| $N_{obs}$ | 115 | | | |
| $F$ test | (27, 88) = 1.41, $p$ = 0.119 | | | |
| $R^2/R^2_{adj}$ | 0.30/0.09 | | | |

**Table S4.** Results of the VAR model with *antimicrobial* difference as the outcome.

| *terms* | *Estimate* | *SE* | *t* | *Pr(>\|t\|)* |
|---|---|---|---|---|
| trend | 0.01 | 0.02 | 0.40 | 0.690 |
| time period [COVID surge vs. baseline (ref.)] | 0.13 | 1.93 | 0.07 | 0.946 |
| | *Lag 1* | | | |
| nanosilver | -0.15 | 0.14 | -1.06 | 0.290 |
| propolis | 0.53 | 0.20 | 2.72 | **0.008** |
| antimicrobial | -0.87 | 0.19 | -4.54 | **< 0.001** |
| antibacterial | 0.27 | 0.17 | 1.56 | 0.122 |
| biomaterials | 0.34 | 0.11 | 3.09 | **0.003** |
| | *Lag 2* | | | |
| nanosilver | -0.14 | 0.17 | -0.82 | 0.412 |
| propolis | 0.17 | 0.19 | 0.89 | 0.373 |
| antimicrobial | -0.49 | 0.21 | -2.28 | **0.025** |
| antibacterial | 0.38 | 0.22 | 1.72 | *0.089* |
| biomaterials | 0.26 | 0.14 | 1.80 | *0.075* |
| | *Lag 3* | | | |
| nanosilver | 0.04 | 0.18 | 0.21 | 0.835 |
| propolis | 0.21 | 0.19 | 1.08 | 0.285 |
| antimicrobial | -0.38 | 0.20 | -1.88 | *0.063* |
| antibacterial | 0.15 | 0.23 | 0.65 | 0.517 |
| biomaterials | -0.17 | 0.14 | -1.17 | 0.245 |
| | *Lag 4* | | | |
| nanosilver | 0.27 | 0.15 | 1.81 | *0.074* |
| propolis | 0.45 | 0.18 | 2.47 | **0.016** |
| antimicrobial | -0.03 | 0.19 | -0.15 | 0.878 |
| antibacterial | -0.29 | 0.21 | -1.42 | 0.160 |
| biomaterials | -0.28 | 0.14 | -1.99 | **0.050** |
| | *Lag 5* | | | |
| nanosilver | 0.24 | 0.12 | 2.04 | **0.044** |
| propolis | 0.48 | 0.19 | 2.51 | **0.014** |
| antimicrobial | 0.00 | 0.15 | 0.00 | 0.997 |
| antibacterial | -0.44 | 0.16 | -2.72 | **0.008** |
| biomaterials | -0.18 | 0.13 | -1.41 | 0.162 |
| $N_{obs}$ | 115 | | | |
| *F* test | (27, 88) = 3.68, $p < 0.001$ | | | |
| $R^2/R^2_{adj}$ | 0.53/0.39 | | | |

**Table S5.** Results of the VAR model with *antibacterial* difference as the outcome.

| *terms* | *Estimate* | *SE* | *t* | *Pr(>\|t\|)* |
|---|---|---|---|---|
| trend | 0.00 | 0.03 | 0.14 | 0.888 |
| time period [COVID surge vs. baseline (ref.)] | -0.27 | 3.08 | -0.09 | 0.930 |
| *Lag 1* | | | | |
| nanosilver | 0.07 | 0.22 | 0.29 | 0.770 |
| propolis | 0.25 | 0.31 | 0.80 | 0.426 |
| antimicrobial | 0.11 | 0.31 | 0.37 | 0.712 |
| antibacterial | -0.67 | 0.27 | -2.45 | **0.016** |
| biomaterials | -0.18 | 0.18 | -1.00 | 0.322 |
| *Lag 2* | | | | |
| nanosilver | -0.05 | 0.27 | -0.20 | 0.845 |
| propolis | 0.18 | 0.31 | 0.59 | 0.558 |
| antimicrobial | 0.06 | 0.34 | 0.17 | 0.868 |
| antibacterial | -0.47 | 0.35 | -1.33 | 0.188 |
| biomaterials | -0.05 | 0.23 | -0.21 | 0.833 |
| *Lag 3* | | | | |
| nanosilver | 0.17 | 0.28 | 0.62 | 0.537 |
| propolis | 0.03 | 0.30 | 0.09 | 0.926 |
| antimicrobial | -0.28 | 0.32 | -0.89 | 0.378 |
| antibacterial | -0.27 | 0.37 | -0.73 | 0.469 |
| biomaterials | -0.07 | 0.23 | -0.33 | 0.743 |
| *Lag 4* | | | | |
| nanosilver | 0.06 | 0.24 | 0.24 | 0.808 |
| propolis | 0.35 | 0.29 | 1.22 | 0.227 |
| antimicrobial | 0.09 | 0.30 | 0.29 | 0.776 |
| antibacterial | -0.41 | 0.33 | -1.26 | 0.211 |
| biomaterials | -0.09 | 0.23 | -0.40 | 0.691 |
| *Lag 5* | | | | |
| nanosilver | 0.15 | 0.19 | 0.78 | 0.439 |
| propolis | 0.42 | 0.30 | 1.39 | 0.168 |
| antimicrobial | -0.02 | 0.24 | -0.08 | 0.935 |
| antibacterial | -0.43 | 0.26 | -1.67 | *0.099* |
| biomaterials | -0.05 | 0.20 | -0.25 | 0.803 |
| $N_{obs}$ | 115 | | | |
| $F$ test | (27, 88) = 1.26, $p$ = 0.209 | | | |
| $R^2/R^2_{adj}$ | 0.28/0.06 | | | |

**Table S6.** Results of the VAR model with *biomaterials* difference as the outcome.

| *terms* | *Estimate* | *SE* | *t* | *Pr(>\|t\|)* |
|---|---|---|---|---|
| trend | 0.00 | 0.02 | 0.05 | 0.961 |
| time period [COVID surge vs. baseline (ref.)] | 0.61 | 2.40 | 0.25 | 0.801 |
| | *Lag 1* | | | |
| nanosilver | -0.22 | 0.18 | -1.23 | 0.224 |
| propolis | 0.42 | 0.24 | 1.71 | *0.091* |
| antimicrobial | -0.55 | 0.24 | -2.31 | **0.023** |
| antibacterial | 0.27 | 0.21 | 1.25 | 0.214 |
| biomaterials | -0.03 | 0.14 | -0.19 | 0.853 |
| | *Lag 2* | | | |
| nanosilver | 0.10 | 0.21 | 0.47 | 0.639 |
| propolis | 0.14 | 0.24 | 0.58 | 0.561 |
| antimicrobial | -0.07 | 0.27 | -0.27 | 0.787 |
| antibacterial | -0.02 | 0.28 | -0.06 | 0.951 |
| biomaterials | -0.12 | 0.18 | -0.68 | 0.501 |
| | *Lag 3* | | | |
| nanosilver | 0.10 | 0.22 | 0.44 | 0.660 |
| propolis | 0.09 | 0.24 | 0.39 | 0.696 |
| antimicrobial | 0.07 | 0.25 | 0.27 | 0.789 |
| antibacterial | -0.15 | 0.29 | -0.52 | 0.603 |
| biomaterials | -0.52 | 0.18 | -2.94 | **0.004** |
| | *Lag 4* | | | |
| nanosilver | 0.40 | 0.19 | 2.15 | **0.034** |
| propolis | 0.44 | 0.23 | 1.94 | *0.055* |
| antimicrobial | -0.28 | 0.23 | -1.20 | 0.235 |
| antibacterial | -0.39 | 0.26 | -1.51 | 0.134 |
| biomaterials | -0.23 | 0.18 | -1.29 | 0.199 |
| | *Lag 5* | | | |
| nanosilver | 0.42 | 0.15 | 2.86 | **0.005** |
| propolis | 0.14 | 0.24 | 0.61 | 0.545 |
| antimicrobial | -0.21 | 0.18 | -1.12 | 0.266 |
| antibacterial | -0.35 | 0.20 | -1.71 | *0.091* |
| biomaterials | -0.13 | 0.16 | -0.80 | 0.424 |
| $N_{obs}$ | 115 | | | |
| $F$ test | (27, 88) = 2.36, $p$ = 0.001 | | | |
| $R^2/R^2_{adj}$ | 0.42/0.24 | | | |

**Table S7.** Results of the causality effects of keyword popularity on other keywords using instantaneous and delayed approaches.

| *Keyword* | *Instant causality* | *Delayed causality* |
|---|---|---|
| Nanosilver | $\chi^2$ (4) = 47.86, $p$ < 0.001 | $F$ (20, 440) = 1.63, $p$ = 0.042 |
| Propolis | $\chi^2$ (4) = 49.97, $p$ < 0.001 | $F$ (20, 440) = 1.24, $p$ = 0.215 |
| Antimicrobial | $\chi^2$ (4) = 47.22, $p$ < 0.001 | $F$ (20, 440) = 1.34, $p$ = 0.151 |
| Antibacterial | $\chi^2$ (4) = 52.93, $p$ < 0.001 | $F$ (20, 440) = 3.00, $p$ < 0.001 |
| Biomaterials | $\chi^2$ (4) = 36.67, $p$ < 0.001 | $F$ (20, 440) = 3.59, $p$ < 0.001 |

**Table S8.** IRF coefficients with *95% CI* according to the impulse and response keywords.

| *Impulse keyword* | *Response keyword* | *Lag order* | | | | | |
|---|---|---|---|---|---|---|---|
| | | *0* | *1* | *2* | *3* | *4* | *5* |
| nanosilver | propolis | 5.03[1]<br>1.29 – 7.96[2] | -1.73<br>-3.39 – 0.29 | -1.18<br>-3.13 – 0.63 | -0.18<br>-1.51 – 0.99 | -0.54<br>-1.78 – 0.64 | 0.11<br>-0.88 – 1.25 |
| nanosilver | antimicrobial | 3.22<br>0.57 – 4.60 | 0.30<br>-0.86 – 1.26 | -0.63<br>-1.88 – 1.07 | 0.21<br>-1.07 – 1.80 | 0.99<br>-0.12 – 2.19 | -0.75<br>-1.67 – 0.61 |
| nanosilver | antibacterial | 7.67<br>1.49 – 12.51 | -2.76<br>-5.12 – 0.30 | -1.68<br>-3.00 – 0.38 | 0.37<br>-0.61 – 1.64 | -0.02<br>-1.39 – 1.08 | 0.26<br>-0.75 – 1.32 |
| nanosilver | biomaterials | -1.21<br>-2.74 – 0.48 | 0.65<br>-1.09 – 2.55 | 0.12<br>-1.43 – 1.79 | 0.23<br>-1.26 – 1.34 | 1.13<br>-0.51 – 2.60 | -0.96<br>-2.58 – 0.70 |
| propolis | nanosilver | 0.00<br>0.00 – 0.00 | 1.89<br>0.41 – 3.75 | -0.59<br>-2.00 – 1.01 | -0.77<br>-2.03 – 0.84 | -0.12<br>-1.73 – 1.23 | 0.36<br>-0.79 – 1.46 |
| propolis | antimicrobial | 1.87<br>0.53 – 2.47 | 1.59<br>0.25 – 2.54 | -0.91<br>-1.90 – 0.19 | -1.55<br>-2.44 - -0.04 | -0.39<br>-1.67 – 0.75 | 1.20<br>-0.23 – 2.15 |
| propolis | antibacterial | 3.03<br>1.03 – 3.64 | -1.07<br>-2.54 – 0.76 | -0.17<br>-1.38 – 1.67 | -1.15<br>-3.17 – 0.88 | 0.60<br>-1.86 – 1.95 | 0.72<br>-0.64 – 2.09 |
| propolis | biomaterials | 1.14<br>-0.33 – 2.52 | 1.32<br>-0.07 – 2.46 | -1.86<br>-2.56 – 0.41 | -0.76<br>-2.11 – 0.53 | -0.11<br>-1.18 – 1.30 | 0.20<br>-1.32 – 1.30 |
| antimicrobial | nanosilver | 0.00<br>0.00 – 0.00 | 0.96<br>-0.26 – 2.02 | -0.74<br>-1.92 – 0.60 | -1.72<br>-3.00 – -0.21 | 0.66<br>-0.55 – 2.16 | -0.27<br>-1.31 – 0.83 |
| antimicrobial | propolis | 0.00<br>0.00 – 0.00 | -0.93<br>-1.66 – 0.33 | 0.03<br>-1.06 – 0.85 | -0.98<br>-1.68 – 0.28 | 0.93<br>-0.31 – 1.62 | -0.65<br>-1.63 – 0.48 |
| antimicrobial | antibacterial | 1.62<br>0.50 – 2.04 | -1.21<br>-2.93 – 0.82 | 0.09<br>-1.38 – 1.84 | -1.75<br>-3.04 – 0.015 | 1.43<br>-0.82 – 2.87 | -0.59<br>-1.76 – 1.06 |
| antimicrobial | biomaterials | 3.62<br>1.61 – 4.46 | -2.17<br>-3.05 – -0.73 | -0.39<br>-1.37 – 0.71 | -1.01<br>-2.02 – 0.49 | -0.96<br>-2.30 – 0.27 | 0.29<br>-0.77 – 1.50 |
| antibacterial | nanosilver | 0.00<br>0.00 – 0.00 | 0.74<br>-0.73 – 1.59 | -1.09<br>-2.31 – 0.47 | -0.23<br>-1.40 – 0.95 | -0.28<br>-1.49 – 1.35 | -0.41<br>-1.56 – 0.78 |
| antibacterial | propolis | 0.00<br>0.00 – 0.00 | -1.29<br>-2.13 – 0.38 | -0.70<br>-1.78 – 0.44 | 0.33<br>-0.95 – 1.27 | 0.06<br>-1.02 – 1.18 | 0.13<br>-0.99 – 1.08 |
| antibacterial | antimicrobial | 0.00<br>0.00 – 0.00 | 0.07<br>-1.05 – 1.09 | -0.27<br>-1.23 – 0.78 | -0.33<br>-1.19 – 0.69 | -0.76<br>-1.67 – 0.43 | -0.64<br>-1.60 – 0.49 |
| antibacterial | biomaterials | -3.02<br>-3.80 – 1.30 | 1.18<br>-0.32 – 2.00 | -1.06<br>-2.14 – 0.65 | 0.66<br>-0.51 – 1.92 | -0.86<br>-1.89 – 0.69 | 0.00<br>-1.41 – 1.22 |
| biomaterials | nanosilver | 0.00<br>0.00 – 0.00 | 0.64<br>-0.85 – 1.91 | -1.34<br>-2.64 – 0.54 | -0.14<br>-1.27 – 1.32 | 0.08<br>-1.75 – 1.58 | 0.47<br>-1.12 – 1.34 |
| biomaterials | propolis | 0.00<br>0.00 – 0.00 | -0.48<br>-1.52 – 0.40 | 0.09<br>-1.16 – 1.00 | 0.04<br>-0.88 – 1.14 | -0.47<br>-1.35 – 0.65 | 0.49<br>-0.66 – 1.54 |
| biomaterials | antimicrobial | 0.00<br>0.00 – 0.00 | 1.83<br>0.62 – 2.70 | -0.87<br>-1.72 – 0.28 | -1.98<br>-2.58 – -0.79 | -1.06<br>-2.01 – 0.11 | 1.16<br>-0.18 – 2.06 |
| biomaterials | antibacterial | 0.00<br>0.00 – 0.00 | -0.94<br>-2.31 – 0.79 | 0.52<br>-1.49 – 2.06 | -0.09<br>-1.59 – 1.62 | -0.58<br>-2.27 – 1.45 | 0.30<br>-1.45 – 1.84 |

[1] coefficient
[2] *95% CI*

**Table S9.** Forecast Error Variance Decomposition (FEVD) contributions at steps 1, 3, and 5.

| *Keyword* | *Step* | *Nanosilver* | *Propolis* | *Antimicrobial* | *Antibacterial* | *Biomaterials* | *Self* |
|---|---|---|---|---|---|---|---|
| Nanosilver | 1 | - | 0.00% | 0.00% | 0.00% | 0.00% | 100.00% |
| | 3 | - | 4.77% | 1.78% | 2.11% | 2.69% | 88.64% |
| | 5 | - | 5.21% | 5.59% | 2.14% | 2.57% | 84.48% |
| Propolis | 1 | 63.76% | - | 0.00% | 0.00% | 0.00% | 36.24% |
| | 3 | 58.60% | - | 1.72% | 4.28% | 0.48% | 34.92% |
| | 5 | 56.07% | - | 5.03% | 4.25% | 0.87% | 33.78% |
| Antimicrobial | 1 | 30.08% | 10.18% | - | 0.00% | 0.00% | 59.74% |
| | 3 | 22.69% | 14.34% | - | 0.16% | 8.58% | 54.23% |
| | 5 | 19.50% | 15.43% | - | 1.24% | 14.97% | 48.86% |
| Antibacterial | 1 | 67.06% | 10.48% | 3.01% | - | 0.00% | 19.45% |
| | 3 | 64.42% | 9.64% | 3.83% | - | 1.08% | 21.03% |
| | 5 | 60.10% | 10.44% | 7.97% | - | 1.30% | 20.19% |
| Biomaterials | 1 | 2.72% | 2.45% | 24.54% | 17.10% | - | 53.19% |
| | 3 | 2.65% | 9.11% | 25.13% | 16.30% | - | 46.82% |
| | 5 | 4.00% | 8.85% | 24.79% | 15.98% | - | 46.39% |

*Note*: Values represent the percentage of forecast error variance explained by each keyword at the specified step. "Self" indicates the proportion explained by the keyword's own shocks. Dashes indicate the keyword's own contribution (not applicable in that row).
